# Estimation of cardiorespiratory fitness in healthy using seismocardiography

**DOI:** 10.1038/s44325-025-00053-x

**Published:** 2025-05-22

**Authors:** S. E. Schmidt, M. T. Hansen, D. S. Karbing, Kasper Sørensen, M. K. Poulsen, T. Rømer, J. J. Struijk, P. Søgaard, J. W. Helge

**Affiliations:** 1 Department of Health Science and Technology, Aalborg University, Aalborg, Denmark; 2 VentriJect Aps, Hellerup, Denmark; 3 Department of Biomedical Sciences, University of Copenhagen, Copenhagen, Denmark; 4 Department of Cardiology, Aalborg University Hospital, Aalborg, Denmark

**Keywords:** Cardiology, Cardiovascular diseases

## Abstract

Cardiorespiratory fitness expressed as maximal oxygen consumption (V̇O_2max_) is a strong predictor of cardiovascular health, but its measurement through cardiopulmonary exercise (CPX) testing is complex and costly. This study develops and validates an algorithm for non-exercise estimation of V̇O_2max_ using seismocardiography (SCG-V̇O_2max_). Data from SCG recordings and CPX tests of 300 subjects were combined into a database, with 83 subjects undergoing repeated sessions. SCG was recorded via a sensitive accelerometer on the lower sternum in a supine position. A machine learning algorithm was trained on data from 221 subjects, with 74 subjects comprising a test set. SCG- V̇O_2max_ (44.8 ± 9.4 ml/min/kg) was comparable to CPX V̇O_2max_ (44.0 ± 10.2 ml/min/kg), with a correlation of *r* = 0.873. Day-to-day variation was low for both methods. SCG-based estimation of V̇O_2max_ is a novel, easy-to-use, and accurate method for assessing cardiorespiratory fitness, with high reproducibility and potential for integration into health evaluations.

## Introduction

Cardiorespiratory fitness (CRF) is an essential part of the individual health status and is strongly related to cardiovascular outcomes including mortality. Recent studies have demonstrated that low fitness level carries a higher mortality risk than any other cardiac risk factors such as smoking, diabetes, hypertension, known cardiac disease, atrial fibrillation, and chronic kidney disease^1^. Furthermore, the health benefit of even minor improvement in CRF is well documented^2^. Thus, the assessment of CRF should be an integrated part of a general health assessment as suggested by the American Heart Association^3^. However, quantifying CRF as V̇O_2max_ through cardiopulmonary exercise (CPX) assessment, which necessitates exercise to the point of exhaustion, is a complex and cumbersome process, rendering it impractical in most clinical settings.

Alternatives to CPX testing include submaximal exercise testing and non-exercise estimation. Submaximal exercise testing encompasses heart rate-based tests like the Aastrand test^4^, the YMCA test^5^ or the V̇O_2max_ estimates from smartwatches such as the Garmin, Fitbit or Apple Watch. Non-exercise testing is an alternative method when exercise testing is not possible, such as during standard clinical health assessments in a primary physician’s office, where exercise equipment is often not available. Non-exercise methods are primarily equation-based models based on demographic data. The approach for non-exercise V̇O_2max_ estimation is most often regression algorithms that use demographic data such as sex, age, height, weight, BMI, resting heart rate, and self-reported physical activity level^6–14^. These methods are limited by mid-range accuracy, that they are not personalized, and that the algorithms relating to self-reported physical activity are subjective.

This paper proposes a novel non-exercise method to predict V̇O_2max_ from a chest-mounted accelerometer while the subject is at rest, based on machine learning of seismocardiography (SCG) signals and demographic data. SCG, the measurement of precordial vibrations using an accelerometer, was first described in 1956 by Mounsey et al.^15^. The SCG waveforms correspond to mechanical events in the cardiac cycle^16,17^. To our knowledge, Libonati et al. were the first to relate SCG time intervals to CRF^18^. The authors used SCG to measure the Tai index, also known as a myocardial performance index, and found a significantly lower myocardial performance index in subjects with the highest fitness level^18^. Sørensen et al. demonstrated a correlation between both time intervals and amplitudes in the SCG signal and the V̇O_2max_ score^19^. The measure with the highest correlation to V̇O_2max_ was the amplitude of the diastolic SCG complex. Shandhi et al. used the wearable sensor to quantify instantaneous V̇O_2_ estimated when doing exercise^20^. The sensor measures SCG and electrocardiogram and by using machine learning predicts current V̇O_2_ consumption.

In this retrospective study, we develop and validate an algorithm for the estimation of relative V̇O_2max_ using SCG (SCG-V̇O_2max_), and in addition, we compare the performance of the SCG-V̇O_2max_ to a state-of-the-art non-exercise algorithm, the FRIEND Equation^14^.

## Results

In total 300 subjects were registered in the database. Four subjects were excluded, two due to missing SCG data, one due to missing ergometer data and one due to hypertrophic cardiomyopathy. In addition, the SCG signal was too weak for the algorithm’s quality control in two recordings leading to the exclusion of one subject. In total, the current analysis includes 295 subjects, where 221 were used for training and 74 were included in the test set. Each subject participated in 1–3 test sessions and between 1-9 recordings were obtained per subject, in total data are included from 458 ergometer sessions and 510 SCG recordings (372 training set and 138 test set), see Table 1. The subjects were 34.8 ± 12.5 years old, (18–72 years) and 134 (45.4%) were female. The average V̇O_2max_ was 43.3 ± 10.5 ml/min/kg. There were no significant differences in demographic variables between the training set and the test set, see Table 1.

**Table 1:** Baseline characteristics and V̇O_2max_ values of the study participants in training and test set

	All	Training set	Test set
n	295	221 (74.9%)	74 (25.1%)
Female (*p* = 0.21)	134 (45.4%)	105 (47.5%)	29 (39.2%)
Age, years (*p* = 0.65)^w^	34.8 ± 12.5 31[25–42]	34.3 ± 11.9 31[25–40.2]	36.2 ± 14.1 31[24–48]
Height, cm (*p* = 0.076)^w^	176 ± 9 177[169–183]	175 ± 9 176[168–183]	178 ± 9 179[171–184]
Weight, kg (*p* = 0.36)	75.2 ± 12.1 75.2[66.8–83.4]	74.8 ± 12.3 74.7[65.5–83.6]	76.4 ± 11.4 76.5[69–82.9]
BMI (*p* = 0.248)^w^	24.2 ± 3.3 23.6[22–25.6]	24.2 ± 3.3 23.6[22–26.0]	24.2 ± 3.2 23.4[22-25.5]
n recordings	510	372 (72.9%)	138 (27.1%)
Recordings per subject (*p* = 0.55)^w^	1.76 ± 2.0 1[1,2]	1.69 ± 1.9 1[1,2]	1.97 ± 2.3 1[1,2]
CPX V̇O_2max_, ml/min/kg (*p* = 0.45))^w^	43.3 ± 10.5 43.6[36.4–51.5]	43.0 ± 10.6 43.7 [36.2–51.3]	44.0 ± 10.2 43.6[37.1–52.1]
SCG-V̇O_2max_, ml/min/kg (*p* = 0.36)^w^	43.4 ± 9.7 43[35.8–50.6]	42.8 ± 9.8 42.7(34.9–50.3)	44.8 ± 9.4 46.8(36.4–51.2)
FRIEND Eq. V̇O_2max_, ml/min/kg (*p* = 0.29)^w^	38.1 ± 8.6 37.7[31.8–45.6]	37.7 ± 8.6 37.2[30.9–44.9]	39 ± 8.7 38.3[33.5–46.5]

Continues variables: mean (±SD) & median [Interquartile range]. Numbers: n (proportion %). ^w^ Wilcoxon rank sum test.

### V̇O_2max_ estimation

**Fig. 1:**
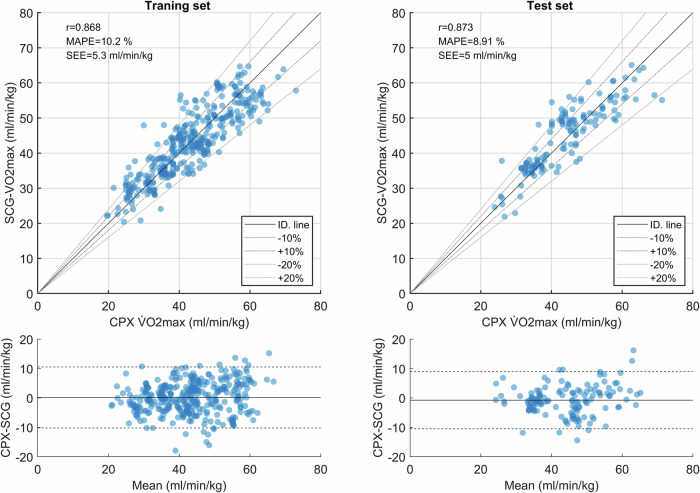
Comparison between Ergometer CPX V̇O2max and SCG-V̇O2max. Top: Scatter plot visualizing the correlation between Ergometer CPX V̇O2max and SCG-V̇O2max in the training and test set. Lower: Bland Altman plots for the training and test set.

In the test set, SCG estimated V̇O_2max_ to 44.8 ± 9.4 ml/min/kg which was comparable to the ergometer CPX V̇O_2max_ at 44.0 ± 10.2 ml/min/kg (*p* = 0.07), see Table 1. The correlation between CPX V̇O_2max_ and the SCG estimate was *r* = 0.868 in the training set and *r* = 0.873 in the test set, Table 2. This corresponds to a coefficient of determination in the training set of *R*^2^ = 0.749 and *R*^2^ = 0.755 in the test set. MAPE was 10.2% in the training set and 8.9% in the test set. The SEE was 5.3 ml/min/kg and 5.0 ml/min/kg, accordingly, and the limits of agreements (LoA) were −10.5 to 10.2 ml/min/kg in the training set and −9.0 to 10.5 ml/min/kg in the test set, see Table 2 & Fig. 1.

**Table 2:** Estimation performance for FRIEND equation and the SCG estimates of V̇O_2max_

	FRIEND equation		SCG-VO_2max_	
	Training set	Test set	Training set	Test set
n (Recordings)	372	138	372	138
Bias (ml/min/kg)	−5.3 [−5.9,-4.6]*	−5 [−6,-3.9]*	−0.16 [−0.7,0.38]	0.77 [−0.07,1.6]
LoA (ml/min/kg)	[−17.9 7.3]	[−17.2 7.3]	[−10.5 10.2]	[−9.0 10.5]
SEE (ml/min/kg)	8.3	8.0	5.3	5.0
MAPE (%)	15 [14,16%]	14.6 [13.1,16.2%]	10.2 [9.4,11%]	8.9 [7.6,10.2%]
r	0.792 [0.75,0.83]	0.79 [0.72,0.85]	0.868 [0.84,0.89]	0.873 [0.83,0.91]
R^2^	0.380 [0.3,0.46]	0.377 [0.25,0.5]	0.749 [0.71,0.79]	0.755 [0.69,0.82]

Estimated value [95% confidence interval of estimate], *Bias significantly different from zero. LoA: limits of agreement.

The FRIEND Equation did underestimate V̇O_2max_ in both datasets (p < 0.001), see Table 2. In the test set, the FRIEND Equation estimated V̇O_2max_ at 39.0 ± 8.7 ml/min/kg. The correlation between FRIEND Equation and CPX V̇O_2max_ was *r* = 0.792 in the training set and *r* = 0.790 in the test set. Corresponding to a coefficient of determination in the training set at *R*^2^ = 0.380 and *R*^2^ = 0.377 in the test set. For the FRIEND Equation MAPE was 15% and 14.6% and SEE was 8.3 ml/min/kg and 8.0 ml/min/kg. Results of the non-SCG score and the Wasserman score can be seen in Supplementary Table 7.

### Reproducibility and tracing of changes in cardiorespiratory fitness level

**Fig. 2:**
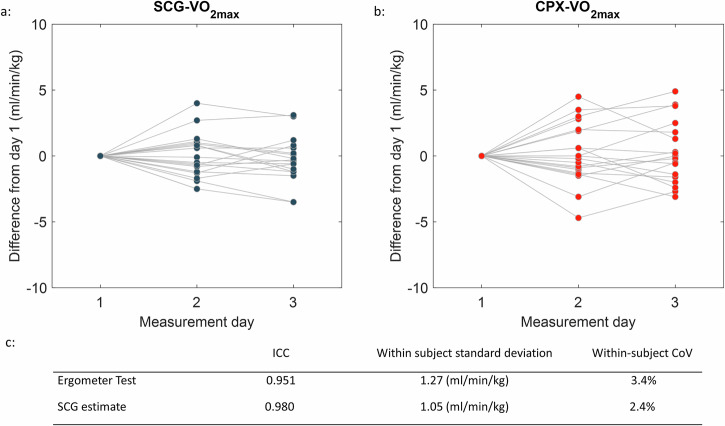
Dato day variations. **a** Variations across 3 measurement days for SCG V̇O_2max_ estimates. **b** Variations across 3 measurement days for ergometer CPX V̇O_2max_. All measurements were taken within 14 days. **c** repeatability statics for the two methods.

Reproducibility was estimated in the 20 subjects that underwent three separate and identical test days within two weeks. The reproducibility of the SCG estimates was higher than the reproducibility of the ergometer CPX measures, Fig. 2. The ICC was 0.98 for the SCG and 0.951 for CPX as the within-subjects standard deviation was 1.05 ml/min/kg in SCG and 1.27 ml/min/kg in CPX. Reproducibility for the FRIEND Equation was not reported since age and sex are two out of three equation parameters that do not change from day to day.

In the database, 63 subjects had repeated recordings with more than 45 days between. CPX V̇O_2max_ increased from 48.1 ± 14.0 ml/min/kg at the first measurement to 50.4 ± 13.9 ml/min/kg in the second measurement (*p* < 0.001), see Table 3. Similarly, the SCG-V̇O_2max_ estimate increased significantly from 47.1 ± 11.9 ml/min/kg to 48.6 ± 11.4 ml/min/kg (p < 0.001). In 66.7% (CI:54-78%) of the subjects the CPX and the SCG estimate agreed on the direction of change. If we excluded subjects with less than 2% change in CPX V̇O_2max_ this agreement increased to 73.1%. (CI: 59–84%). The FRIEND Equation did not change significantly as the estimated V̇O_2max_ was 42.9 ± 8.9 ml/min/kg at the first measurement and 43.0 ± 8.45 ml/min/kg at the second measurement (*p* = 0.24).

**Table 3:** Two measurements of V̇O_2max_ in 63 subjects with more than 45 days between the measurements

	Initial measurement	Follow up (>45 days later)	Difference	Significance
Ergometer Test (ml/min/kg)	48.1 ± 14.0	50.4 ± 13.9	2.28 ± 3.0	*p* < 0.001
**SCG estimate (ml/min/kg)**	47.1 ± 11.9	48.6 ± 11.4	1.45 ± 2.5	*p* < 0.001
FRIEND Eq. (ml/min/kg)	42.9 ± 8.9	43 ± 8.45	0.11 ± 0.8	*p* = 0.24

### Classification of cardiorespiratory fitness level

The percentile limits 0–25%, 25–75% and 75–100% from the FRIEND cohort^21^ were used to divide subjects into CRF groups low, medium, and high. 14 (2.7%) recordings were classified as low, 220 (43.1%) as medium, and 276 (54.1%) as high. Table 4 shows SCG-V̇O_2max_ estimates’ capacity to classify subjects in the same three CRF groups as CPX. The SCG classifies 77.3% of subjects in the correct CRF group and Cohen’s Kappa was 0.56, while the FRIEND Equation classifies 59.0% of subjects in the correct CRF group and Cohen’s Kappa was 0.27 for the FRIEND Equation.

**Table 4:** Classification of the subject’s cardiorespiratory fitness (CRF) level into low, medium, and high accruing to the limits defined by the FRIEND cohort^21^

SCG-V̇O_2max_						FRIEND Eq.					
		Predicted CRF group			Total			Predicted CRF group			Total
		Low	Medium	High				Low	Medium	High	
**True CRF group**	Low	6 (1.2%)	8 (1.6%)	0 (0.0%)	14 (2,8%)	**True CRF group**	Low	6 (1.2%)	8 (1.6%)	0 (0.0%)	14 (2,8%)
	Medium	6 (1.2%)	156 (30.6%)	58 (11.2%)	220 (43,1%)		Medium	2 (1.6%)	216 (42.4%)	2 (0.4%)	220 (43,1%)
	High	0 (0%)	44 (8,5%)	232 (45.5%)	276 (54,1%)		High	0 (0.0%)	197 (38.6%)	79 (15.5%)	276 (54,1%)
Total		12 (2.4%)	208 (40.8%)	290 (56.9%)	510 (100%)	Total		8 (1.6%)	421 (82.6%)	81 (15.9%)	510 (100%)

## Discussion

This study demonstrates the potential of a novel method for non-exercise assessment of cardiorespiratory fitness. The SCG method requires less than one minute’s recording of SCG for estimating the cardiac function. In conjunction with heart rate, age, sex, height, and weight the cardiac function measures are used to estimate V̇O_2max_. The SCG-based estimation of V̇O_2max_ has a high correlation (*r* = 0.873) to CPX V̇O_2max_ and through analysis of repeated measures we found a high level of reproducibility of the method.

The rationale for the estimation of SCG-V̇O_2max_ at rest is based on Fick’s equation in which the peak cardiac output is a major factor in V̇O_2max_. Peak cardiac output depends on the maximal heart rate and the maximal stroke volume, which depends highly on diastolic function. Gerche et al. found that cardio anatomical measures, such as end-diastolic volume and ventricular mass obtained using magnetic resonance imaging at rest were superior to functional measures obtained during exercises in the prediction of V̇O_2max_^22^. Agam et al. examined the correlation between diastolic SCG measures and echocardiographic measures and found a high correlation between the diastolic relaxation parameter é and the diastolic SCG peak-to-peak variable which we here named ${{Dia}}_{{pp}}$^23^. Other studies have linked systolic SCG measures with cardiac contractility^24–26^, thereby the SCG-V̇O_2max_ estimate builds on measures of both diastolic relaxation and systolic contraction. The V̇O_2max_ variance explained by SCG-V̇O_2max_ was *R*^2^ = 0.755 in the test set, while this was only *R*^2^ = 0.64 for the non-SCG score, see supplementary Table S7. This demonstrates the unique contribution of the SCG components. The SCG-V̇O_2max_ method relies on a cloud-based algorithm meaning that the algorithm can continuously be updated as more data become available for training of the algorithm. The improvements for the prior algorithms can be seen in Supplementary Fig. 2. Figure 1 demonstrates heteroscedasticity in the estimation error, with higher estimation errors observed in subjects with higherV̇O_2max_ compared to those with lower V̇O_2max_. This may reflect that central circulatory factors predominantly predict V̇O_2max_ in the normal and low CRF groups, while other factors, such as peripheral factors, may have a greater impact on V̇O_2max_ in subjects with very high CRF^27^.

The current study demonstrates the superiority of the SCG-V̇O_2max_ estimate compared to the FRIEND Equations estimate of V̇O_2max_. The SCG-V̇O_2max_ had a lower bias, higher correlation, lower SEE and lower percent-wise error in MAPE. In the context of health assessment, the most important aspect is that this performance increase led to significantly improved classification, 77.3% vs 59.0%, in the correct CRF levels groups. In general, the FRIEND Equations classified the majority of subjects (82.6%) in the median CRF group, while only 43.1% were classified in the median CRF group according to CPX V̇O_2max_, Table 4. This highlights the lack of personalised estimation of statistical models like the FRIEND Equations.

In addition, the SCG-V̇O_2max_ traced the group average improvements of V̇O_2max_ in the subjects with repeated measurements recorded more than 45 days apart. However, the agreement between CPX and SCG-V̇O_2max_ at the individual level was moderate, since the methods agreed change in only 73.1% of cases where V̇O_2max_ changed more than 2%, however, it is worth noting that repeated measures of the CPX test showed a significant day-to-day variation, Fig. 1.

Smartwatches, such as the Polar smartwatch, are an alternative to equation-based non-exercise V̇O_2max_ estimates. However, according to Molina-Garcia et al.^28^, the reported limits of agreement of non-exercise estimated V̇O_2max_ by watches were -13.97 to 17.41 ml/kg/min. These are considerably wider limits than the limits of agreement reported in the current study for both the FRIEND Equations and the SCG-V̇O_2max_, as shown in Table 2.

When some exercise is feasible, submaximal tests are an alternative to the non-exercise tests. Here the heart rate at one or two load levels of exercise is used to extrapolate to a V̇O_2max_. Commonly used tests are the Aastrand and the YMCA tests. Beekley et al. validated the YMCA test and found a correlation at *r* = 0.79 between CPX V̇O_2max_ and the YMCA V̇O_2max_ estimate^29^. Their measure of total error corresponds to our SEE measure. The total error of the estimate of the YMCA test was 18.5 ml/min/kg, which is considerably higher than the SEE of the SCG-V̇O_2max_ at 5.0 ml/min/kg. Similar performance was identified for the Aastrand test with *r* = 0.71-0.78 and SEE 6.2-9.7 ml/min/kg^6^. Comparisons of prediction metrics across studies should be done with caution, but the result of the discussed studies points towards the superiority of SCG-V̇O_2max_ compared to other commonly used clinical submaximal tests.

Molina-Garcia et al.^28^ evaluated exercise-based estimated V̇O_2max_ by smartwatches such as Garmin, Fitbit, or Apple Watch, finding limits of agreement at -9.9 to 9.7 ml/min/kg. These are comparable to the performance of SCG-V̇O_2max_ in the current study, which has a limit of agreement at −9.0 to 10.5 ml/min/kg. However, these methods necessitate multiple exercise sessions, rendering them inconvenient for on-site testing.

SCG-V̇O_2max_ is a method for point of care estimation of V̇O_2max_. SCG recordings as short as 42 s are sufficient for accurate estimation of V̇O_2max_ and with a cloud processing time of around 30 s, a recording can be made in 2–3 min, in addition, the method is easy to use and at low cost. These properties could facilitate SCG-V̇O_2max_ to become a biomarker for use in regular health assessment, which could lead to improved health in the general population and public savings.

The current study is strengthened by the pooling of multiple studies with a balanced proportion of sex and age ranging from 18–72 years. However, 54.1% of the subjects belonged to the high-fitness group, as defined by the FRIEND study, and only 2.8% to the low-fitness group, meaning that the current population is relatively fit. In addition, all participants were recruited in Denmark where most subjects are Caucasians, and all subjects were without known cardiovascular disease and other known major diseases. Significant international heterogeneity in CRF is well-documented^30,31^. For example, some nationalities, such as Norwegians, typically score higher V̇O_2max_ values compared to others. These differences may not be fully captured by non-exercise test algorithms based on demographic data. Since the current method is partly based on demographic measures, it might be subject to the same population bias. Future studies could validate SCG-V̇O_2max_ performance across different nationalities. Further studies in new patient groups will enable the potential of SCG-V̇O_2max_ monitoring of patients undergoing diagnosis and rehabilitation. It is also important to note that since SCG-V̇O_2max_ estimates are derived from cardiac signal analysis, the respiratory and metabolic components of V̇O_2max_ are not considered in the SCG-V̇O_2max_ estimation.

SCG-based estimation of V̇O_2max_ is a novel, easy-to-use, and accurate estimation method of CRF in healthy participants, with a high level of reproducibility. In our opinion SCG-V̇O_2max_ can facilitate that cardiorespiratory fitness becomes an integrated part of modern health assessment.

## Methods

### Study population

SCG recordings and results from CPX testing of V̇O_2max_ in subjects from six clinical studies were combined in a database. The studies were two from Aalborg University (Aalborg university study 1 and 2) and four from the University of Copenhagen (University of Copenhagen study 1, University of Copenhagen study 2, a Sensor Validation Study and a Football study). Aalborg university study 1 included 23 females undergoing an 8-week CrossFit training program^19^. SCG and ergometer CPX V̇O_2max_ were obtained prior and posterior to the CrossFit training period. Aalborg university study 2 was a supplement to Aalborg university study 2 where SCG and ergometer CPX V̇O_2max_ were obtained from 10 males^19^. Follow-up measurement was conducted one year later. The aim of the University of Copenhagen study 1 and 2^28,32^ was to collect data for algorithm development and validation and the studies included in total 200 subjects undergoing ergometer CPX. The Sensor Validation Study aimed to compare the medical device, Seismofit, against the prototype used in earlier studies and to study the reproducibility of the method ^33^. Repeated measures with SCG recording and ergometer CPX were conducted on three separate days within two weeks in 20 subjects. It was assumed that V̇O_2max_ would be steady throughout the two weeks. SCG was recorded with both the prototype device and the Seismofit device on each test day. In the Football study^34,35^, the SCG and treadmill CPX V̇O_2max_ were obtained during the pre-season 2021 from 47 Danish sub-elite football players and an additional 8 weeks later in the spring season. Supplementary Tables 1 and 2 in the supplementary materials include details on all study subjects.

In all studies CPX V̇O_2max_ was obtained with grade exercise tests and pulmonary gas exchange rate was continuously obtained via breath by breath analyse using 10 sec automatic sampling averages by the equipment software. V̇O_2max_ was determined as the highest V̇O_2_ measured during 30 consecutive seconds, the detailed protocols and acceptance criteria’s, can be found in supplementary Table 3.

In all studies, the inclusion criteria were that the age should be above 18 years. The exclusion criteria differed across the studies, but pregnancy and cardiovascular disease are exclusion criteria in the current analysis. All studies were conducted in accordance with the Declaration of Helsinki. Informed consent was obtained from all individual subjects included in the studies. The local scientific ethics committees approved the research protocols for each study.

### SCG recordings

SCG recordings were obtained using a sensitive accelerometer, located on the lower sternum at the Xiphoid protrusion and subjects were placed in a supine position. In the first studies (The Aalborg university studies, the University of Copenhagen study 1 and 2, and the Sensor Validation Study) a prototype sensor was used for data collection. A low noise accelerometer (Silicon Designs 1521-002, Silicon Designs, Kirkland, Washington, USA) was embedded in a 3D printed housing and data were recorded using an Iworx data acquisition box (IWORX, Dover, New Hampshire, USA) with a sample rate at 5.000 samples per second in combination with a 3-lead electrocardiogram.

In the Sensor Validation Study^33^ and the Football study SCG was recorded with the medical device, Seismofit (VentriJect Aps, Hellerup, Denmark). The Seismofit device is a CE-marked medical device for the estimation of V̇O_2max_. The Seismofit device records 42 s of SCG and the sample rate was 1.100 samples per second. After recording, the data are transferred to a cloud solution where algorithms analyse the recordings and estimate a V̇O_2max_ score. Analyses in the current study are based on retrospective analyses of the collected recordings. To avoid overfitting the dataset was divided into a training and a test set. The test set was composed of subjects randomly selected from the University of Copenhagen Study 2, the Sensor Validation Study and the Football study.

### Pre-processing of the SCG signal

**Fig. 3:**
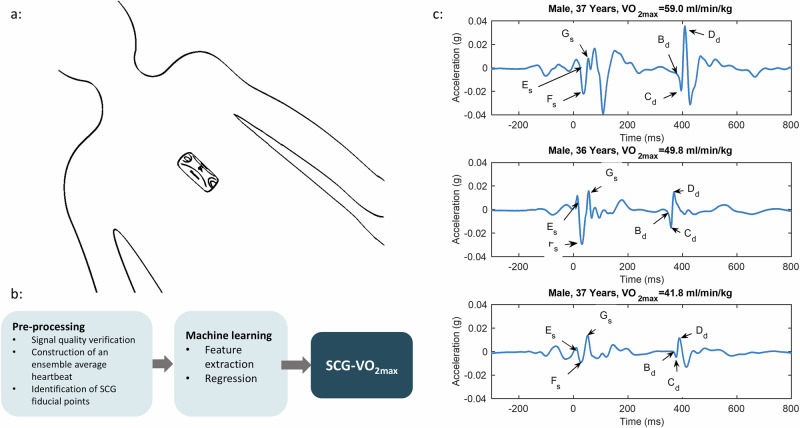
Sensor and signal overview. **a** The SCG sensor is mounted at the lower sternum. **b** Overview of the algorithm steps. **c** Ensemble average SCG waveforms from three subjects with different levels of fitness. The Es fiducial point marks the closing of the mitral valve, Gs correlates to the opening of the aortic valve, and Bd to the closure of the aortic valve. Fiducials point Cd is the first minima after B_d_ and D_d_ the first peak.

As SCG measures accelerations, SCG is inherently sensitive to movement artefacts. Movement artefacts are often most dominant at the beginning and end of the recording. Therefore, we excluded the first and the last segments of the recordings if the variance was more than 2 times the variance of the remaining recording. To optimise feature extraction the SCG was segmented into individual heartbeats using a duration-dependent Markow model as described in Schmidt et al.^36^. Before further analysis, an algorithm checks the heart rhythm for arrhythmias that could potentially cause algorithmic errors. No cases of arrhythmia were present in the current population. The SCG fiducial points^16^, see Fig. 3, were identified automatically using a prototherian algorithm (VentriJect Aps, Hellerup, Denmark).

### Development of regression model

We selected features from both the diastolic and systolic SCG. Sørensen et al. found the diastolic peak to peak value from the Cd point to the Dd point (${{Dia}}_{{pp}}$) showed the highest degree of correlation to V̇O_2max_ among a wide range of SCG measures^19^.

In addition to the peak-to-peak measure, we quantified the morphology of the SCG (${DiaMorpSex}$) by principal component analysis of all ensemble average SCG waves in the training set. As in Hansen et al.^28^ we combined the first two principal components using a linear regression into one Diastolic Morphology Measure. Since we observed differences between sexes, we trained the model in both females and males. Thereby we developed a sex-specific Diastolic Morphology measure.

Since the systolic complex morphology is highly variable from subject to subject we quantified the Systolic SCG using frequency spectrum analysis (${SysSpec}$). The systolic complex was quantified using a power spectrum analysis of the systolic SCG. This spectrum was further composed into a single systolic measure using a linear regression of the first three principal components.

In addition to the SCG measures described above we included resting heart rate, age, sex, height and weight in the linear regression model. See formula (1).

1 $$\begin{array}{rcl}\begin{array}{c}SCG{-{VO}}_{2{\max }}\,=\,{\omega }_{0}\,+\,{\omega }_{1}Sex\,+\,{\omega }_{2}Age\,+\,{\omega }_{3}Weight\,+\,{\omega }_{4}Height\\ \qquad\qquad\qquad\qquad\qquad+\,{\omega }_{5}RR\,+\,{{\omega }_{5}{Dia}}_{{pp}}\,+\,{\omega }_{6}DiaMorpSex\,+\,{\omega }_{7}SysSpec\end{array}\end{array}$$

The regression weights (ω) were found by least square optimization in the training set. To avoid those subjects with multiple recordings over-influenced the algorithm, the recordings were weighted according to the number of repeated measurements per subject. The correlation between CPX V̇O_2max_ and the individual features can be seen in Supplementary Table 6.

### Comparison to non-exercise equation estimation of V̇O_2max_

Using the large Fitness Registry and the Importance of Exercise National Database (FRIEND) cohort (*n* = 7.759) Myers et al. developed a non-exercise algorithm for V̇O_2max_ estimation based on age, sex and weight^14^. We consider this algorithm the state of art algorithm for non-exercise estimation of V̇O_2max_. We compared the estimation performance between SCG-V̇O_2max_ and the FRIEND Equation and we compare how the two approaches classify subjects into CRF groups, defined as the lower 25% percentile, the 25–75% percentile and the upper 75% percentile according to the FRIEND’s cohort and based on relative V̇O_2max_^21^. To determine the influence of the SCG components we also constructed a non-SCG score from the dataset, based only on age, sex, height and weight. We also estimated V̇O_2max_ using the Wasserman equation^11^.

### Statistical analysis

Variables are expressed as mean (±standard deviation (SD)). Categorical variables are reported as frequencies (percentages). The unpaired/paired student t-test was used for comparison between continuous variables when normal distribution was not precluded by the Shapiro-Wilk Test. Wilcoxon rank sum test was used in cases of distributions deviating from the normal distribution defined by Shapiro-Wilk Test. The chi-square test was used for comparison between categorical variables. Pearson correlation was used to analyse correlations between V̇O_2max_ and estimated V̇O_2max_. Prediction error was quantified using mean average percentage error (MAPE) or standard error of estimate (SEE). SEE was estimated as

2 $${SEE}=\sqrt{\frac{\sum {(Y-\dot{Y})}^{2}}{N-2}}\begin{array}{c}{\rm{Y}}={\mathrm{CPX\; VO}}_{2max}\\ \,\dot{Y}={\mathrm{EstimateVO}}_{2max}\end{array}$$

The Coefficient of determination (R^2^) was estimated as one minus the sum of squares of residuals divided by the total sum of squares^37^. We analysed repeatability by the intra-class correlation (ICC), within-subject coefficient of variation (CoV), and within-subjects standard deviation. We compare classification into three fitness groups using accuracy and Cohen’s kappa.

## Supplementary information

Supplementary materials

## Data Availability

The study data was provided from Aalborg University, VentriJect and Copenhagen University in anonymised form. The processed data used for statistical analysis that support the findings of this study are available from the corresponding author, upon reasonable request. Due to intellectual property and confidentiality raw SCG recordings and the Seismofit algorithm are unavailable for sharing.
