# Supplementary material for: Estimation of cardiorespiratory fitness in healthy using seismocardiography
Source: NPJ Cardiovasc Health. 2025 May 22;2:16. doi: 10.1038/s44325-025-00053-x (PMC12912316; doi:10.1038/s44325-025-00053-x)
Supplement: Supplementary file 1 — Supplementary materials [file 44325_2025_53_MOESM1_ESM.pdf]

Supplementary materials for Estimation of cardiac fitness using seismocardiography

Supplementary Table 1. Clinical studies in the study database.

Study name	Description	Number of Subj.	Sensor	Reference to publication	Local ethical committee number
Aalborg university study 1	Females undergoing an 8-week CrossFit training program. Ergometer CPX and SCG pre and post training<br>AIM: Proof of concept	23	Prototype	S1	N-20160034
Aalborg university study 2	Males recruited at Aalborg university, DK. Ergometer CPX and SCG measured twice with one year.<br>AIM: Proof of concept	10	Prototype	S1	N-20160034
University of Copenhagen study 1	Subjects recruited at the University of Copenhagen, DK<br>AIM: Data collection & validation	100	Prototype	S2	H-17008748
University of Copenhagen study 2	Subjects recruited at the University of Copenhagen<br>AIM: Data collection & validation	100	Prototype	S3	H-18031350
Sensor validation study	Repeated Measurement (Ergometer CPX & SCG) on 3 different days in 20 subjects. Subjects recruited at the University of Copenhagen<br>60 prototype recordings and 120 Seismofit recordings<br>AIM: Study of repeatability. Study of equivalence between prototype & Seismofit Performance validation	20	Prototype & Seismofit	S4	H-19081375
Football study	Repeated measurement in football players with more than 8 weeks between. Subjects recruited at the University of Copenhagen<br>AIM: Performance study in highly fit subjects	47	Seismofit	S5	H-19043654

S1. K, S. *et al.* A Clinical Method for Estimation of VO2max Using Seismocardiography. *International journal of sports medicine* (2020).

s2. Hansen, M. *et al.* Determination of Maximal Oxygen Uptake Using Seismocardiography at Rest. in *Computing in Cardiology* vol. 48 (2021).

S3 Hansen MT, Husted KLS, Fogelstrom M, Romer T, Schmidt SE, Sorensen K, et al. Accuracy of a Clinical Applicable Method for Prediction of VO 2max Using Seismocardiography. *Int J Sports Med. 2022;*

S4 Thunestvedt Hansen, M. *et al.* Validity and reliability of seismocardiography for the estimation of cardiorespiratory fitness. *Cardiovascular Digital Health Journal 2023*

S5 Rømer T, Hansen MT, Lange KK, et al. Peak fat oxidation, peak oxygen uptake, and running performance increase during pre-season in sub-elite male football players. *Scand J Med Sci Sports*. 2024;

Supplementary Table 2: Demographic data in the individual studies.

	Total	Aalborg university 1	Aalborg university 2	University of Copenhagen 1	University of Copenhagen 2	Sensor validation	Football
n	295	23	9	99	97	20	47
Female (p=0.21)	134 (45.4%)	23 (100%)	0 (0%)	50 (50.5%)	51 (52.6%)	10 (50%)	0 (0%)
Age, years (p=0.27)	34.8±12.5	36.8±5.15	41.2±13.7	30.3±6.97	41.3±13.8	46.4±16.7	23.8±3.92
Height, cm (p=0.07)	176±9	169±6	185±4	175±9	175±9	176±9	183±5
Weight, kg (p=0.36)	75.2±12.1	77.9±15.1	83.9±12.7	72.6±11.6	75.4±13.1	73.5±11.5	78.0±7.6
BMI (p=0.83)	24.2±3.3	27.4±5.3	24.5±3.3	23.7±2.6	24.6±3.6	23.5±1.9	23.2±1.7
n recordings	510	39	16	99	97	172	87
Recordings per subject (p=0.29)	1.73±2.0	1.74±0.5	1.78±0.4	1±0	1±0	8.6±0.5	1.91±0.3
Reference VO_{2Max} (ml/min/kg)	43.3±10.5	28±4.4	39.4±8.4	46.4±7.1	39.3±8.1	39.5±6.2	57.3±5.1
Included in the Test set	138 (27.9%)	0 (0%)	0 (0%)	0 (0%)	49 (50.5%)	57 (33.1%)	32 (36.8%)

Supplementary Table 3. CPX protocols as utilized in the studies.

Study name	CPX equipment	Ramp protocol	Acceptance Criteria for VO2max
Aalborg university study 1 [S1]	Vyntus® CPX metabolic cart (Jaeger, Carefusion, Hoechberg, Germany)<br><br>Ergometer: Ergomedic Peak Bike 894, (Monark Exercise AB, Sweden)	**Cycle ergometer exercise**. 1-minute intervals starting at 104 W load, with increase in load of 24 W between intervals. Subjects were asked to keep a consistent cadence at 80 revolutions per minute (RPM) to keep the load consistent.	The protocol continued until the subject was unable to maintain the 80 RPM due to exhaustion. Respiratory exchange rate >1.15 was used as indication that maximal effort was reached.[S1]
Aalborg university study 2 [S1]	Vyntus® CPX metabolic cart (Jaeger, Carefusion, Hoechberg, Germany)<br><br>Ergometer: Ergomedic Peak Bike 894, (Monark Exercise AB, Sweden)	**Cycle ergometer exercise**. 1-minute intervals starting at 104 W load, after 2 minutes the load was increased to 160, and there after an increase in load of 40 W between intervals. Subjects were asked to keep a consistent cadence at 80 revolutions per minute (RPM) to keep the load consistent.	The protocol continued until the subject was unable to maintain the 80 RPM due to exhaustion. Respiratory exchange rate >1.15 was used as indication that maximal effort was reached.[S1]
University of Copenhagen study 1 [S2]	Quark CPET, (Cosmed, Rome, Italy)<br><br>Ergometer: Monark 839E, (Monark Exercise AB, Sweden)	**Cycle ergometer exercise.** 5-minute warm-up at 75W participants performed a graded exercise test with 25W increments every minute until voluntary exhaustion on a cycle ergometer	The V̇ O2max criteria was O2 levelling off and a respiratory exchange ratio > 1.15.
University of Copenhagen study 2 [S3]	Quark CPET, (Cosmed, Rome, Italy)<br><br>Ergometer: Corival (Lode, Netherlands)	**Cycle ergometer exercise**. The exercise protocol started with a five-minute warm-up at 50 W for women and 100 W for men and was followed by a 25 W increment every minute until voluntary exhaustion.	1. criteria: Plateau in $V.O_2$, defined as <2.1 $mL \cdot min^{-1} \cdot kg^{-1}$ increment in $V.O_2$ with increasing workload.<br><br>2. criteria: A respiratory exchange ratio > 1.10, HRmax within 10 bpm of age-predicted HRmax (220-age)
Sensor validation study [S4]	Quark CPET, (Cosmed, Rome, Italy)<br><br>Ergometer: Monark 839E, (Monark Exercise AB, Sweden)	**Cycle ergometer exercise.** The warm-up and initial test workload of the CPET protocol differed between sexes and training status and increased by 20 and 25 W every minute for women and men, respectively.	1. criteria: Plateau in $V.O_2$, defined as <2.1 $mL \cdot min^{-1} \cdot kg^{-1}$ increment in $V.O_2$ with increasing workload.<br><br>2. criteria: Respiratory exchange ratio >1.10
Football study [S5]	Quark CPET, (Cosmed, Rome, Italy)<br><br>Treadmill: Woodway Pro XL,(Woodway Inc, USA)	**Treadmill exercise**. Protocol started with a 5-min warm-up at 6 km·h−1 with an incline of 1%. Then it increased in velocitywith 2 km·h−1 every third minute with the incline at 1% until thesteady state respiratory exchange ratio (RER) exceeded 0.95 . Subsequently, the velocity was constant and the incline increased by2% every minute until voluntary exhaustion. P	1. criteria: Plateau in $V.O_2$, defined as <2 $mL \cdot min^{-1} \cdot kg^{-1}$ increment in $V.O_2$ with increasing workload.<br><br>2. criteria: RER exceeding 1.10

Supplementary Note 1: Equivalence between prototype and Seismofit device

In the sensor validation study, $\dot{V}O_{2max}$ was estimated using both a prototype and the medical device Seismofit in 20 subjects, three times each, to demonstrate equivalence between the prototype and Seismofit®. The correlation between the prototype and Seismofit was r=0.970, and the difference between Seismofit and the prototype was 0.02±1.6 ml/min/kg (see Figure S1). The correlation to ergometer $\dot{V}O_{2max}$ was r=0.829 for Seismofit and r=0.831 for the prototype. Therefore, we consider the performance of the two devices to be equivalent.

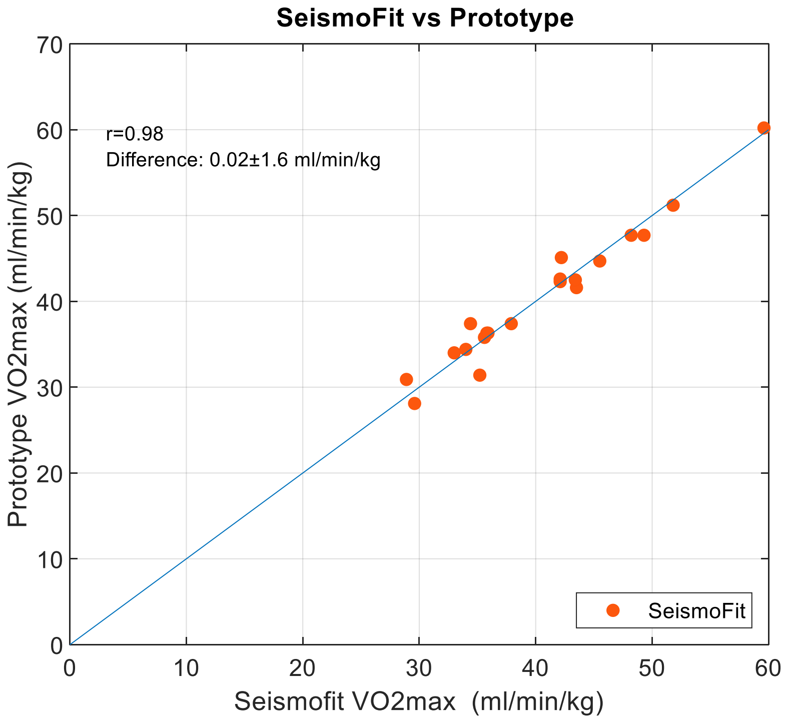

Supplementary Figure 1. Equivalence between prototype and Seismofit device. Comparison between VO_{2max} estimates based on the prototype and the Seismofit device.

Supplementary Note 2: Development in algorithm performance

The SCG-$\dot{V}O_{2max}$ algorithm has undergone a significant development, as more data has become available for development. Figure S2 shows the performance of the recent algorithms in the current test set. None of the test subjects has been used for the development of the algorithms. The algorithm described in the current study is version 4.7.

Tables S3 and S4 describe the configurations of the different algorithms.

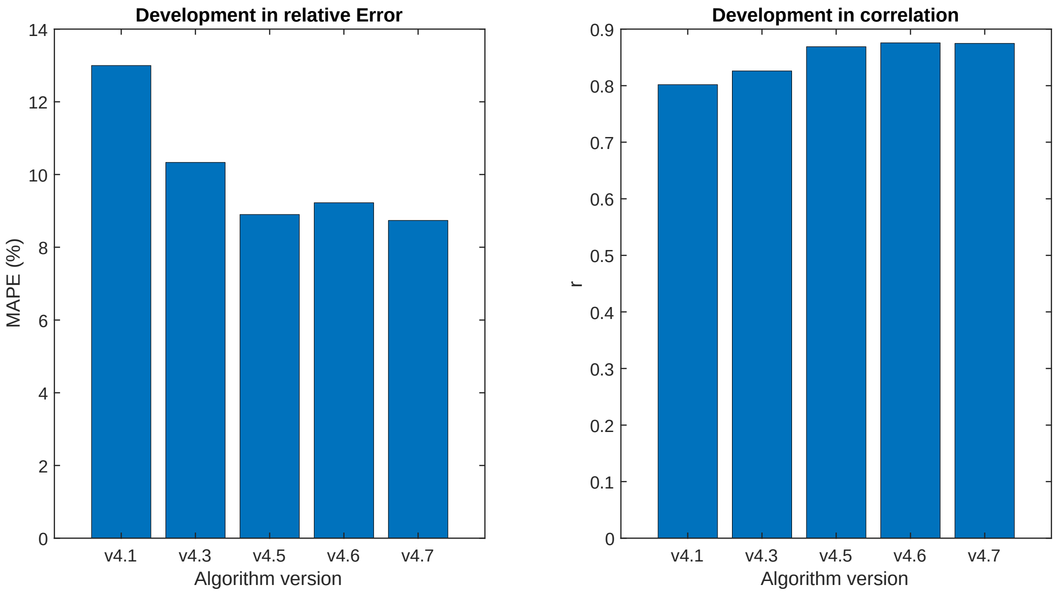

Supplementary Figure 2. Performance of algorithm versions. Left: MAPE of the recent SCG-VO$_{2max}$ algorithms in the current test set. Right: correlation of the recent SCG-VO$_{2max}$ algorithms in the current test set.

Supplementary Table 4: Linear regressions used in the different algorithm versions.

Algorithm version	Features included in the linear regressions
4.1	1+DiaSpec+ DiaMorp + RR + SysSpec+ FRIEND_Algo
4.3	1+DiaSpec+ DiaMorp + RR + SysSpec+ FRIEND_Algo + amp_D$_d$ + amp_K$_S$ + SYSRV$_{STD}$ + amp_RF + amp_AO_min
4.3	1+DiaSpec+ DiaMorp + RR + SysSpec+ FRIEND_Algo + amp_D$_d$ + amp_F$_s$ + SYSRV$_{STD}$ + amp_L$_s$
4.5	1+Sex + Age + Weight + Height + RR + Dia$_{pp}$ + DiaMorp$_{Sex}$ + SysSpec
4.6	1+Sex + Age + Weight + Height + RR + LogDia$_{pp}$ + DiaMorps$_{ex}$+ SysSpec + DiaSpec$_{sex}$ + amp_D$_s$ + LogDia$_{pp_100Hz}$ + ampE$_{s_40Hz}$ + SysSTD_$_{150Hz}$
4.7	1+Sex + Age + Weight + Height + RR + Dia$_{pp}$ + DiaMorp$_{Sex}$ + SysSpec

Supplementary Table 5: Description of features in Table 4

**Feature name**	**Feature description**
AGE	Age (years)
SEX	Gender (Female=0 & Male=1)
Weight	Weight (kg)
Height	Height (cm)
FRIEND_Algo:	VO_{2max} predicted using a demographic-based algorithm (Sex, age and body weight)<br>Myers et al. A Reference Equation for Normal Standards for VO2 Max: Analysis from the Fitness Registry and the Importance of Exercise National Database (FRIEND Registry)
RR	The average duration of a heartbeat
Dia_{pp}	Peak to peak amplitude in SCG diastolic complex from the C_d to the D_d fiducial points
$LogDia_{pp}$	Same as above but log transformed
amp_D_d	Amplitude of D_d fiducial point
amp_K_S	Amplitude of K_S fiducial point
amp_L_s	Amplitude of Ls fiducial point
amp_Ds	Amplitude of Ds fiducial point
ampE_s_40Hz	Amplitude of Es fiducial point after high pass filtering (40 Hz)
Sys_{STD}_150Hz	The standard deviation of the systolic complex after high pass filtering (150 Hz)
$SYSRV_{STD}$	Variation in systolic duration
SysSpec	Frequency of the average SCG systolic complex quantified using PCA.
DiaSpec	Frequency of the average SCG diastolic complex quantified using PCA.
$DiaSpec_{Sex}$	Frequency of the average SCG diastolic complex quantified using PCA the model is gender specific
DiaMorp	Morphology of the average SCG diastolic complex quantified using PCA.
$DiaMorp_{Sex}$	Morphology of the average SCG diastolic complex quantified using PCA. the model is gender specific

Supplementary Table 6: Pearson correlation between $\dot{V}O_{2max}$ and features

	Training set			Test set		
	**All**	**Female**	**Male**	**All**	**Female**	**Male**
**N**	372	175	197	138	53	85
Sex (Male=1)	0.55			0.58		
Age	-0.49	-0.36	-0.64	-0.59	-0.57	-0.67
Weight	-0.05	-0.56	-0.40	0.10	-0.35	-0.37
Height	0.44	0.00	0.02	0.52	0.23	0.18
RR	0.31	0.24	0.24	0.15	0.42	0.05
Diapp	0.66	0.67	0.65	0.75	0.41	0.77
SysSpec	0.50	0.58	0.44	0.45	0.39	0.61
$DiaMorp_{sex}$	0.66	0.41	0.45	0.69	0.43	0.48

Supplementary Table 7: Estimation performance of the non-SCG score and the Wasserman score.

	**Training set**	**Test set**
**Non-SCG score[1]**		
n	372	138
Bias (ml/min/kg)	0.33 (-0.29,0.95)	0.93 (-0.08,1.9)
MAPE (%)	11.9 (10.9,12.9%)	11.8 (10.4,13.3%)
r	0.818 (0.78,0.85)	0.808 (0.74,0.86)
SEE (ml/min/kg)	6.1	6.1
R^2	0.67	0.64
**Wasserman[2]**		
Bias (ml/min/kg)	-7.1 (-7.7,-6.4)*	-6.4 (-7.4,-5.3)*
MAPE (%)	17.8 (16.7,18.9%)	16.4 (14.7,18.1%)
r	0.795 (0.75,0.83)	0.807 (0.74,0.86)
SEE (ml/min/kg)	9.6	8.8
R^2	0.18	0.25

1: Score developed in the current dataset, but excluding SCG data: VO2max~age+sex+Heiaght +Weight 2: Wasserman, Karlman, et al. "Principles of exercise testing and interpretation." Journal of Cardiopulmonary Rehabilitation and Prevention 7.4 (1987)
